# Development of machine learning models for chronic fatigue prediction in granulomatosis with polyangiitis

**DOI:** 10.1186/s42358-025-00482-3

**Published:** 2025-10-09

**Authors:** Alexandre Moura dos Santos, Samuel Katsuyuki Shinjo

**Affiliations:** Division of Rheumatology, Faculdade de Medicina da Universidade de Sao Paulo, Sao Paulo, Brazil

**Keywords:** Granulomatosis with polyangiitis, Fatigue, Machine learning, Vasculitis

## Abstract

**Background:**

Chronic fatigue severely compromises the quality of life in patients with granulomatosis with polyangiitis (GPA). Traditional diagnostic methods are often time-consuming, relying on clinical expertise and detailed questionnaires. This study aimed to develop a machine learning model capable of predicting chronic fatigue in GPA patients based on clinical data, with a particular focus on improving diagnostic capacity in regions with limited access to specialists.

**Methods:**

This cross-sectional study collected data on fatigue (measured by the Modified Fatigue Impact Scale, MFIS), functional ability (Health Assessment Questionnaire, HAQ), disease activity (Birmingham Vasculitis Activity Score, BVAS), comorbidities, medication use, physical activity (International Physical Activity Questionnaire - Short Form, IPAQ-SF), and demographic characteristics. Four machine learning algorithms—logistic regression, decision tree, random forest, and extreme gradient boosting—were assessed using a 70/30 train-test split. Model performance was evaluated using area under the curve (AUC), accuracy, F1 score, recall, and precision. Statistical comparisons were performed using Welch’s t-test and the Wilcoxon-Mann–Whitney U test for continuous variables, while the chi-square test or Fisher’s exact test was applied to categorical variables, with significance set at *P* < 0.05. All analyses were conducted using R version 4.4.1 for Windows.

**Results:**

Forty-five patients were assessed: 62.2% were female, with a median BMI of 27.72 kg/m² (23.2–30.1), a median age of 55.5 years, and a median disease duration of 12.0 years (6.0–17.0). Fatigue was reported by 20 patients (MFIS score ≥ 38), and seven patients (15.5%) had active disease according to the BVAS, which was similar between the fatigued and no fatigued groups (*P* > 0.05). The fatigued group had more acute-phase reactants and prednisone use (*P* < 0.05). The tree-based models achieved an AUC of approximately 0.80, outperforming the other models.

**Conclusion:**

Tree-based models demonstrated superior predictive performance in identifying chronic fatigue. The Random Forest model, in particular, highlighted higher disability in activities of daily living (HAQ), older age, and longer disease duration as key predictors. Although the models performed well, additional data and incorporation of clinically relevant variables may further enhance predictive accuracy. Patients with GPA who experienced fatigue showed higher glucocorticoid use and elevated acute-phase reactants, despite similar levels of disease activity, suggesting mechanisms beyond inflammation. Machine learning shows strong potential as a clinical tool for fatigue identification, especially in settings with limited access to specialist care.

**Trial registration:**

Universal Trial Number (UTN): U1111-1271-6003; Brazilian Clinical Trials Registry (ReBEC): RBR-9n4z2hh. Registration date: January 18, 2022, and Plataforma Brasil (CAAE # 41762820.1.0000.0068).

## Background

Granulomatosis with polyangiitis (GPA) is a rare, multisystemic disorder characterized by primary systemic vasculitis associated with anti-neutrophil cytoplasmic antibodies (ANCA) that primarily target small blood vessels [1]. GPA is characterized by vascular lesions with granulomatous and necrotizing features, primarily affecting the upper and lower respiratory tracts as well as the kidneys [2, 3].

Recent studies have demonstrated that, in addition to the complexity of symptoms and prognoses, the rarity of GPA, with a global annual incidence of approximately 9.0 cases per year, poses a significant challenge in the treatment and study of this disease. In the United Kingdom, the incidence is slightly higher than the global average; however, in countries such as Japan and Italy, it is approximately 2.45 cases per year. In the Americas, the average incidence is 11.4 cases per year, with specific data from Argentina showing a lower annual incidence of 9.0 cases per year. To date, there are no specific data available for Brazil, but it is believed that the incidence in Brazil is similar to the general findings across the Americas [4]. GPA accounts for 16% of all vasculitis cases in the Southeast Region of Brazil, with a higher prevalence in women (59.5%). The female-to-male ratio is approximately 1.5:1.0 [5].

Patients with GPA experience reduced quality of life due to various impairments and sequelae and often suffer from chronic pain and fatigue [6, 7]. Fatigue is a diagnostic challenge owing to its multifactorial nature and is prevalent in 50%-80% of patients with rheumatic diseases. It is considered the primary contributor to reduced quality of life and subsequent limitations in daily activities and is emerging as a key complaint among patients with ANCA-associated vasculitis [6, 8–11].

Owing to its clinical diagnosis, which relies primarily on self-report questionnaires and is often subjective in nature, diagnosing fatigue depends heavily on physicians’ knowledge of fatigue and familiarity with these questionnaires. This can make diagnosis challenging for less specialized physicians. Additionally, the presence of fatigue in patients with active disease activity can further complicate accurate assessment of this symptom.

Given the complex nature of questionnaires in daily clinical practice, where evaluating data for decision-making can be challenging, the utilization of machine learning (ML) represents an emerging field. It harnesses the computational power required to assimilate information and reveals intricate patterns that may elude human perception. This capacity significantly augments the ability to predict events on the basis of previously supplied data, mirroring the diagnostic process undertaken by a physician who interprets signs and symptoms to reach a conclusive diagnosis [12].

Therefore, our study aimed to use ML tools for optimal GPA classification, specifically for the presence of chronic fatigue. We plan to employ straightforward algorithms and low-complexity clinical data tailored to the practices of rheumatologists. Second, we aim to evaluate the accuracy and efficiency of algorithms for prediction with small sample sizes, which are common in rare disease studies, such as those in rheumatology.

## Methods

### Study design

This cross-sectional study involved interviews with patients with GPA who fulfilled the American College of Rheumatology/European League Against Rheumatism (ACR/EULAR) 2022 classification criteria [3]. This study was conducted at a tertiary hospital in March 2022 and December 2023. The study adhered to all guidelines for research involving human subjects following the principles of the Helsinki Treaty [12] and was part of a larger research initiative approved by the local Research Ethics Committee. All the data were collected during routine hospital visits via a structured form under the supervision of an experienced rheumatologist (SKS).

Sensitive data were safeguarded and deidentified according to data protection laws. Data collection and storage were performed via the REDCap^®^ system to ensure overall protection of data integrity and privacy [13]. All patients signed an informed consent form [14].

### Questionnaires and assessment

The following variables were collected/generated, including demographic, clinical, and laboratory data, as well as questionnaire responses: sex, age, weight, height, body mass index (BMI), ethnicity, marital status, disease duration, presence of comorbidities (e.g., dyslipidemia, systemic arterial hypertension, diabetes mellitus, cardiovascular diseases, and acute myocardial infarction), immunosuppressant and immunobiological use, glucocorticoid use, daily dose, use of antihypertensive agents, use of statins, serum C-reactive protein (CRP) levels, erythrocyte sedimentation rate (ESR), and systemic blood pressure (mmHg). Disease activity was assessed using the Birmingham Vasculitis Activity Score (BVAS) version 3 [15, 16].

Fatigue was assessed using the Modified Fatigue Impact Scale (MFIS) [17, 18], with fatigue defined as ≥ 38 points. Activities of daily living were assessed via the Health Assessment Questionnaire (HAQ) [19, 20] and International Physical Activity Questionnaire Short Form (IPAQ-SF) [21].

Individuals with a confirmed diagnosis of fibromyalgia or those using medications that act on the central nervous system, which could interfere with the diagnosis or assessments in the present study, were excluded [22].

### Statistical analysis

Quantitative variables are reported as medians and interquartile ranges [25th–75th percentiles], whereas qualitative variables are presented as relative frequencies (%) and absolute frequencies (n). For the initial comparison between patients with and without fatigue, the Welch *t* test was used for normally distributed data with homoscedasticity, whereas the Wilcoxon Mann‒Whitney U test was employed when normal distribution assumptions were not met. The associations between qualitative variables were investigated via either the chi-square test or Fisher’s exact test, depending on the sample size and distribution. Statistical significance was set at *P* < 0.05 [23]. Analyses were conducted in R 4.4.1 for Windows.

### Machine learning procedures

The data were organized in Tidy format, adhering to the principle of one variable per column and one observation per row [24]. Corrections were made to address errors and units of measurement, and new variables, such as BMI, disease duration, and subscores from the original questionnaires, were calculated. To address missing data (affecting less than 5% of observations), we employed the median for quantitative variables and the mode for categorical variables. One-hot encoding was applied to categorical variables, and Z score normalization was selectively implemented for algorithms that demonstrated this need. These steps were aimed at enhancing the consistency and quality of the data for subsequent analyses [25].

After preprocessing, we split the data into training (70%) and testing (30%) sets via random sampling without replacement [26].

The Boruta algorithm was used to identify the most relevant variables (features) for prediction. It operates by executing multiple models via random forest (RF) and then assessing which variables consistently produce the best predictions across these models. This process categorizes variables into three distinct groups: important, not important, and uncertain. In doing so, Boruta effectively handles the selection of primary variables for the model [27, 28].

First, we employed binary logistic regression (LR), a robust algorithm, to model the relationships between fatigue and several independent variables in the GPA. This model, developed linearly with maximum likelihood parameter estimation, was refined through the selection of clinically and laboratory-relevant variables under the guidance of specialized rheumatologists [29, 30]. This model was implemented via the “glm” function of the stats package in R.

Second, we implemented the decision tree (DT) algorithm via the “rpart” function from the “rpart” package in R. We employed the same variables and data split into training and testing sets as previously described. To assess the model performance and address potential overfitting, we performed a 10-fold cross-validation.

We also utilized the RF algorithm proposed by Breiman [31], implemented in the “randomForest” package using its respective function. This algorithm leverages the power of averaging predictions from multiple trees, thereby improving predictions. These algorithms demonstrate greater predictive ability in datasets with more variables (features) than in observations do, as in our case. Our configuration included 200 DTs and a minimum terminal node size of five, utilizing the variables selected previously [31, 32].

Finally, we employed Xtreme Gradient Boosting (XB), an algorithm that addresses classification and regression problems. It is based on the boosting concept in which models are sequentially created to correct previous errors. It also incorporates penalties in the loss function to prevent overfitting, handles missing values, and increases processing speed by executing the algorithm in parallel [33, 34].

To evaluate the performance of the model, we utilized a comprehensive set of ML metrics, including accuracy, area under the curve (AUC), F1 score, recall, and precision. This diverse range of metrics allows for a thorough assessment of the predictive capabilities of models and facilitates informed comparisons between different models. Furthermore, we demonstrated the importance of the variables for every model, thus enhancing our understanding of the prediction process [35].

Owing to the limited number of patients with GPA and the primary focus of this study on prediction, we chose not to calculate the sample size in advance. The methodology used to develop predictive models in ML fundamentally differs from traditional statistical paradigms. Rather than testing a specific hypothesis with purely statistical bias, the objective here is to develop a robust predictive model capable of generalizing new data. This approach allows for a more practical and clinically relevant evaluation, ensuring the applicability and effectiveness of the predictive results. ML procedures were conducted in R 4.4.1 for Windows. (R Core Team, Vienna, Austria) [36].

## Results

A total of 45 patients with GPA were interviewed during outpatient follow-up, with a median age of 55.5 (43.8–63.0) years and a disease duration of 12.0 (6.0–17.0) years. The median BMI was 27.7 (23.2–30.1) kg/m^2^. Moreover, 62.2% were females, 69% were White, and 55.6% were married.

Most patients used glucocorticoids and immunosuppressants; however, most did not show disease activity according to the BVAS (Table 1). No patients in the cohort received treatment with infliximab or tocilizumab.

**Table 1:** General characteristics and Pharmacological treatment in total sample and fatigue subgroups

	GPA (*n* = 45)	GPA-NF (*n* = 25)	GPA-F (*n* = 20)	*P*-Value
				GPA-F vs. GPA-NF
Age (year)	55.0 (43.0–63.0)	54.0 (44.0–64.0)	57.0 (41.0–61.0)	0.567
Body mass index (kg/m²)	26.7 (23.2–30.1)	27.8 (23.2–28.9)	26.2 (23.5–31.8)	0.653
Disease duration (years)	12.0 (6.0–17.0)	9.0 (5.0–13.0)	16.5 (7.0–23.0)	0.042
**Acute-phase reactants**				
C-reactive protein (mg/L)	3.4 (1.3–6.6)	2.8 (1.1–4.1)	4.9 (2.8-8.0)	0.017
Erythrocyte sedimentation rate (mm/1st hour)	8.5 (5.0-22.5)	5.0 (5.0–9.0)	16.0 (5.7–24.2)	0.024
**Disease Activity n** (%)	7 (15.6)	3 (12.0)	4 (20.0)	0.682
**Drug treatment**				
Use of prednisone n (%)	25 (55.6)	12 (48.0)	13 (65.0)	0.367
Prednisone dose (mg/day)	5.0 (0.0–20.0)	2.5 (0.0–5.0)	17.5 (0.0–25.0)	0.030
Use of immunosuppressants and immunobiologics n (%)	33 (73.3)	18 (72.0)	15 (75.0)	> 0.999
Methotrexate n (%)	9 (20.0)	5 (20.0)	4 (20.0)	> 0.999
Azathioprine n (%)	14 (31.1)	10 (40.0)	4 (20.0)	0.202
Mycophenolate mofetil n (%)	4 (8.9)	1 (4.0)	3 (15.0)	0.309
Leflunomide n (%)	1 (2.2)	0	1 (5.0)	0.444
Rituximab n (%)	9 (20.0)	5 (20.0)	4 (20.0)	> 0.999

Disease activity was assessed using the Birmingham Vasculitis Activity Score version 3 (BVAS v.3). Continuous variables are expressed as median (Q1–Q3), and categorical variables as number (percentage). GPA: granulomatosis with polyangiitis; GPA-NF: granulomatosis with polyangiitis without fatigue; GPA-F: granulomatosis with polyangiitis with fatigue. Statistical significance was set at *p* < 0.05

Six patients (13.3%) were current smokers or had used tobacco within the last six months. Furthermore, 28 patients (62.2%) had systemic arterial hypertension, 15 patients (33.3%) had dyslipidemia, and eight patients (17.8%) had diabetes mellitus (Table 2). Moreover, seven patients (15.6%) had a previous diagnosis of cardiovascular disease, three (6.7%) had a history of stroke (Table 2), and none of the participants had experienced an acute myocardial infarction.

Fatigue was reported by 20 patients (45.0%), with the majority also presenting a low level of physical activity. However, notably, they exhibited a commendable capacity to perform activities of daily living (Table 3).

When comparing patients with fatigue (GPA-F) and those without fatigue (GPA-NF), there was a balance between age and BMI. However, the GPA-F group had a longer diagnosis time and higher values of acute-phase reactants (ESR and CRP) (*P* < 0.05), without differences in disease activity prevalence according to the BVAS. Moreover, the patients consumed more daily prednisone (Table 1). The comorbidities and habits of patients in both subgroups were comparable (Table 2).

Patients in the GPA-F group exhibited reduced activities of daily living. However, there were no significant differences in physical activity levels. Ultimately, these patients had worse fatigue scores and lower values across all the domains (Table 3).

**Table 2:** Comorbidities, habits, and treatments in total sample and fatigue subgroups

	GPA (*n* = 45)	GPA-NF (*n* = 25)	GPA-F (*n* = 20)	*P*-Value
				GPA-F vs. GPA-NF
**Comorbidities**				
Dyslipidemia n (%)	15 (33.3)	7 (28.0)	8 (40.0)	0.527
Systemic arterial hypertension n (%)	28 (62.2)	16 (64.0)	12 (60.0)	> 0.999
Diabetes mellitus n (%)	8 (17.8)	3 (12.0)	5 (25.0)	0.435
Stroke n (%)	3 (6.7)	1 (4.0)	2 (10.0)	0.577
**Medications for comorbidities**				
Use of statins (%)	15 (33.3)	7 (28.0)	8 (40.0)	0.527
Use of antihypertensives (%)	23 (51.1)	13 (52.0)	10 (50.0)	> 0.999
Use of more than one antihypertensive (%)	7 (15.6)	2 (8.0)	5 (25.0)	0.214
**Habits**				
Smoking n (%)	6 (13.3)	5 (20.0)	1 (5.0)	0.204

GPA: granulomatosis with polyangiitis; GPA-NF: granulomatosis with polyangiitis without fatigue; GPA-F: granulomatosis with polyangiitis with fatigue. Categorical variables as number (percentage). Statistical significance was set at *p* < 0.05

**Table 3:** Physical activity and chronic fatigue in total sample and fatigue subgroups

	GPA (*n* = 45)	GPA-NF (*n* = 25)	GPA-F (*n* = 20)	*P*-Value
				GPA-F vs. GPA-NF
**Activities of daily living**				
HAQ score (range: 0.00–3.00)	0.10 (0.00-0.50)	0.00 (0.00-0.10)	0.40 (0.10–0.90)	0.002
**Physical activity**				
METs per week	786.0 (198.0-1360.0)	720.0 (198.0-1224.0)	888.0 (229.5-1549.5)	0.864
**Level of physical activity**				
Low n (%)	24 (53.3)	13 (52.0)	11 (55.0)	> 0.999
Moderate n (%)	17 (37.8)	8 (32.0)	9 (45.0)	0.537
High n (%)	4 (8.9)	4 (16.0)	0	0.117
**Chronic fatigue**				
Fatigue by MFIS (score ≥ 38 points) n (%)	20 (45.5)	0	20 (100)	< 0.001
MFIS- Total score (range: 0–84)	36.0 (21.0–50.0)	22.0 (9.0–28.0)	54.5 (41.7–64.0)	< 0.001
MFIS- Physical domain (range: 0–36)	19.0 (9.0–26.0)	9.0 (4.0–16.0)	26.5 (23.7–30.0)	< 0.001
MFIS- Cognitive domain (range: 0–40)	14.0 (8.0–22.0)	9.0 (4.0–13.0)	23.5 (19.5-27.25)	< 0.001
MFIS- Psychosocial domain (range: 0–8)	4.0 (2.0–6.0)	2.0 (1.0–4.0)	6.0 (5.0-7.25)	< 0.001

GPA: granulomatosis with polyangiitis; GPA-NF: granulomatosis with polyangiitis without fatigue; GPA-F: granulomatosis with polyangiitis with fatigue. Continuous variables are expressed as median (Q1–Q3), and categorical variables as number (percentage). Statistical significance was set at *p* < 0.05

We conducted a correlation analysis between the MFIS score and quantitative variables, revealing a significant positive correlation with the HAQ score (rho = 0.422,*P* = 0.004), the CRP level (rho = 0.362, *P* = 0.014), and disease duration (rho = 0.304, *P* = 0.042). Furthermore, a significant correlation was identified between the HAQ score and age (rho = 0.352, *P* = 0.018).

### Model results

Seventeen variables were selected via the Boruta algorithm on the basis of the RF architecture; 1000 trees were important and uncertain for predicting all the models. These selected variables are listed below: “disease_duration,” “age,” “sex_male,” “BMI,” “CRP,” “ESR,” “disease_activity,” “systemic_blood_pressure,” “dyslipidemia_1,” “diabetes_2,” “haq_score,” “ipac_score,” “prednisone_dose” “immunosuppressant_1,” “marital_single,” ethnicity_white,” and “marital_others.” These variables are dummy-coded (0 and 1), except for “haq_escore,” “disease_duration,” “age”, “BMI,” “CRP,” and “ESR”.

The data were randomly allocated to a training set of 31 patients and a test set of 14 patients. The variable “fatigue” (presence or absence) was separated along with other variables that could provide information about the predicted variable. We used a cutoff of 0.45 (45%) in all the models after analyzing the best prediction among all the models.

**Fig. 1:**
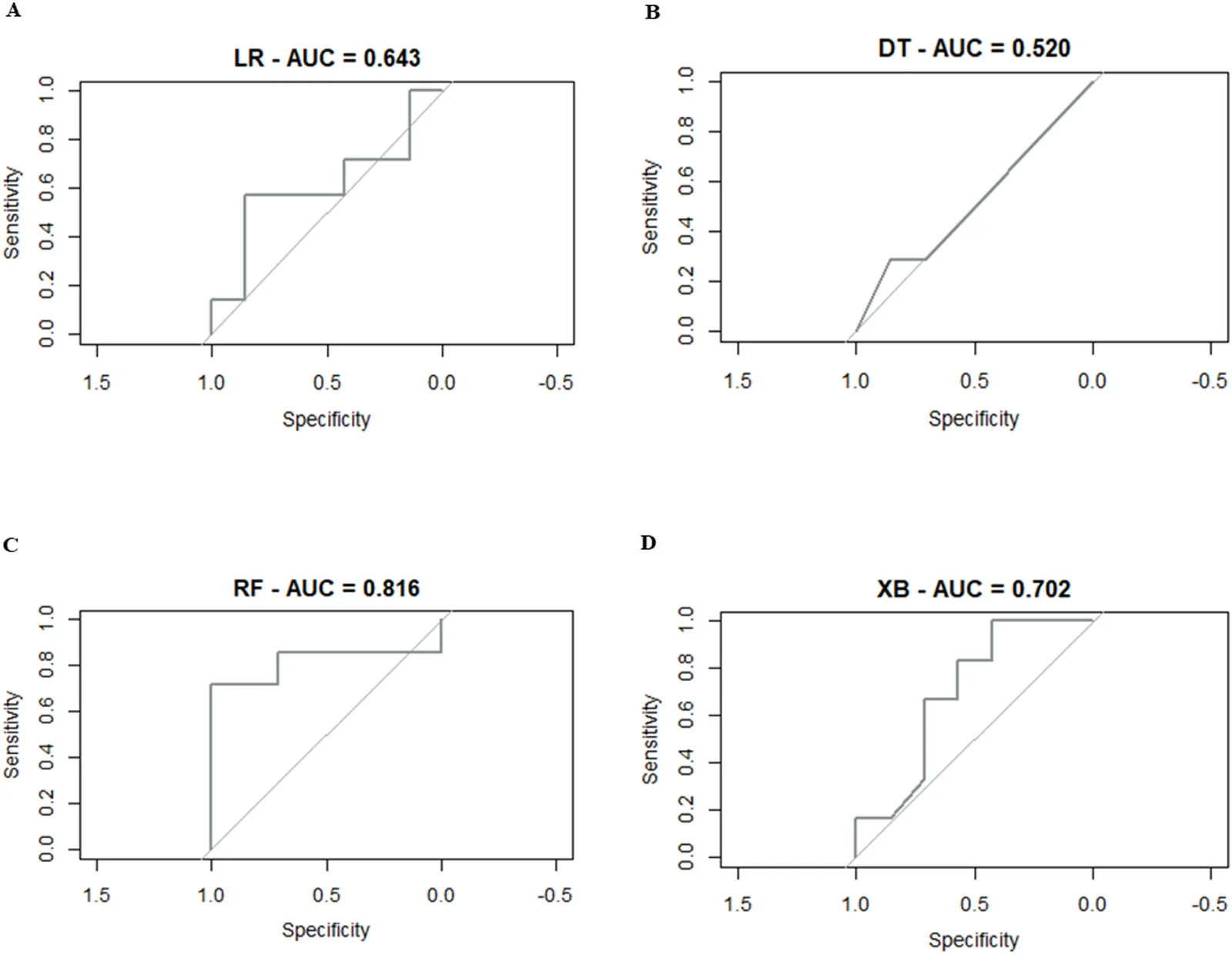
Area under the curve of the models on the test set. AUC: Area Under the Curve, DT: Decision Tree, LR: Logistic Regression (binary), RF: Random Forest, XB: Extreme Gradient Boosting

**Fig. 2:**
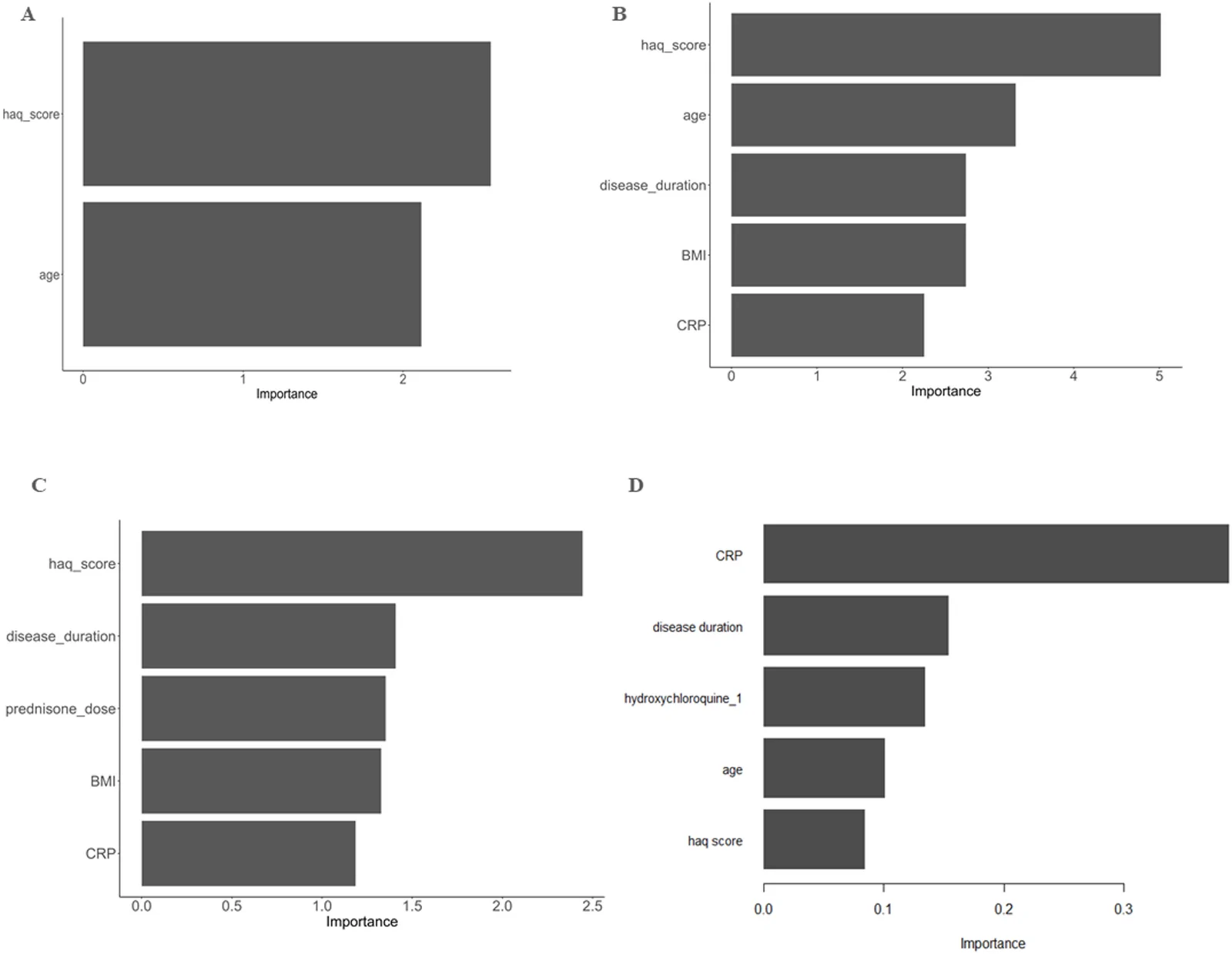
Variable importance of the models on the test set. BMI: Body Mass Index, CRP: C-Reactive Protein, hydroxychloroquine_1: Hydroxychloroquine use

The initial model evaluated the LR, resulting in an accuracy of 0.357, F1-Score of 0.400, recall of 0.428, and precision of 0.376 for the test set. The AUC was 0.643 (Fig. 1A). The two most important variables identified were the HAQ score and prednisone dose (Fig. 2A).

The DT model achieved an accuracy of 0.571, an F1-Score of 0.666, recall of 0.857, and precision of 0.545. Additionally, the AUC was 0.520 (Fig. 1B). The most important variables identified were the HAQ score, age, and disease duration (Fig. 2B).

Compared to all other models, the RF model demonstrated superior predictive performance, achieving an accuracy of 0.786, an F1-Score of 0.800, recall of 0.857, and precision of 0.750. Additionally, the AUC was 0.816 (Fig. 1C). The most important variables for the model were the HAQ score, age, and disease duration (Fig. 2C).

Conversely, the XB model demonstrated an overall accuracy of 0.692, an F1-Score of 0.600, recall of 0.428, and precision of 0.999 on the test set, with an AUC of 0.702 (Fig. 1D). The most relevant variables for prediction were the CRP level, disease duration, and hydroxychloroquine use (Fig. 2D).

## Discussion

This is the first study to classify GPA patients regarding the presence of chronic fatigue using common clinical variables with an ML approach, thus strengthening a new field of research and artificial intelligence in rheumatology diagnosis.

Approximately half of the evaluated patients exhibited chronic fatigue and reduced physical activity levels similar to those described by dos Santos et al. [11] in patients with Takayasu’s arteritis, corroborating the findings in patients with ANCA-associated vasculitis [8].

Fatigue is associated with a reduction in quality of life and increased mortality in the general population [6, 9]. Its development can be influenced by various triggers, such as inflammation or other stressors, disease duration, and genetic and biopsychosocial factors (e.g., anxiety, stress, depression, and physical inactivity) [37].

These factors also contribute to exercise intolerance, worsening sleep quality, muscle pain, and headaches, often overlapping with typical symptoms of rheumatic diseases, specifically vasculitis, including reduced memory, concentration, joint pain, nonrestorative sleep, throat inflammation, and persistence of these complex symptoms for a minimum of six months. These criteria collectively highlight the intricate nature of chronic fatigue, emphasizing its multifaceted manifestations in clinical contexts [38].

Basu et al. [39] also demonstrated differential levels of activity in different brain regions when functional magnetic resonance imaging was used. Specifically, increased activation was observed in the right thalamus, left paracentral lobule, and left medial frontal gyrus in GPA patients who did not experience fatigue compared with those with fatigue. Furthermore, when GPA patients were compared with the general population, greater activation was found in the general population. The regions with significantly greater activation in the control group were the left precentral gyrus, right superior frontal gyrus, right inferior frontal gyrus, and right cingulate gyrus. This pattern of activation suggests the potential dysregulation of neural hubs in patients with fatigue, indicating the inhibition of cortical areas, which may reflect a central nervous system mechanism.

In the present study, seven patients exhibited disease activity with similar prevalence rates between the groups. This finding demonstrates that the potentially relevant factors for the onset of fatigue do not necessarily determine the presence of disease activity. Although patients with fatigue have higher acute-phase reactant values, these values are generally low [40].

Furthermore, in certain cases, the presence of chronic fatigue can complicate the differentiation between patients with active disease and those in remission or the coexistence of activity and chronic fatigue. This ambiguity may contribute to the inappropriate use of glucocorticoids and immunosuppressants, leading to increased side effects from their use, as also observed in our data [41].

We used the MFIS questionnaire to assess fatigue, similar to the approach used by dos Santos et al. [11], and observed a significant correlation with the Fatigue Severity Scale. Thus, the selection was based on the utilization of subdomains, enabling a more nuanced understanding of the fatigue characteristics.

Chronic fatigue is typically evaluated via specific self-report questionnaires, such as patient-reported outcomes. Nevertheless, the effective application and selection of these tools necessitate a profound understanding of fatigue, and rheumatologists are often unfamiliar with their use. Moreover, most of these questionnaires were lengthy, contributing to an extended duration of consultation. Consequently, this can potentially limit the time available to address other clinical demands during consultations, such as medication management and disease activity assessment [42]. On the other hand, Nagy-Szakal et al. [43] have been searching for potential biomarkers with the aid of ML to enable diagnosis and a future therapeutic target, thus improving the lives of millions of patients.

On the basis of this premise, we propose the use of supervised algorithms that utilize information collected traditionally in interviews conducted by rheumatologists, enabling simple identification of the presence of chronic fatigue, thereby facilitating the treatment and diagnosis of chronic fatigue.

In ML, two primary types of algorithms are prominent: supervised and unsupervised algorithms. Supervised models are trained via labeled data to discern patterns and make predictions or to classify new data. Examples of these models include regression and classification models. Conversely, unsupervised models operate on unlabeled data, aiming to identify inherent patterns, clusters, or associations within the data without generating explicit predictions. Examples of such models include clustering algorithms and dimensionality reduction techniques. Both play distinct roles, with supervised models concentrating on predictions and unsupervised models focusing on the underlying structure of the data [44].

With respect to supervised learning models, LR has been traditionally employed for prediction tasks in the healthcare domain. These models provide accurate predictions [45].

In recent decades, tree-based models have garnered significant attention within the realm of healthcare algorithms owing to their ease of implementation and remarkable accuracy in classifying healthcare outcomes, such as the presence of diseases or specific symptoms. This characteristic makes them particularly well suited for application in smaller datasets, which are often encountered in the early stages of research or when dealing with rarer rheumatic conditions [29, 46].

In our study, we found better predictions via two tree-based models, RF and XB, with RF performing better, as also reported in the literature [47]. These models exhibit considerable value in terms of their quality metrics. With larger samples, we could uncover even more relevant values, thus enhancing the predictive capacity. It is well established that more training data often lead to better results. However, another critical factor for prediction quality is the inclusion of more relevant variables.

The models revealed the importance of variables, including low-complexity variables, such as CRP, age, disease duration, prednisone dose, and HAQ score.

These variables corroborate the mechanisms of chronic fatigue in rheumatic diseases proposed by Davies [37]. However, the prednisone dose led us to presume that fatigue may compromise the correct prescription of medications and an accurate understanding of disease activity, given that there was no difference between patients in remission and those with active disease in our study.

Among the most important variables, the inclusion of the HAQ highlights its crucial role in assisting the diagnosis of chronic fatigue. Given its good acceptance by clinicians and easy application, it should be widely used. This finding also indicates the need for further investigations into chronic fatigue.

A key limitation of our study was the limited availability of training data and potentially low-quality variables. However, initiatives are underway to address this challenge, such as the establishment of large patient registries exemplified by the National Data Bank for rheumatic diseases [48].

## Conclusions

In conclusion, all three machine learning models demonstrated satisfactory predictive performance in assessing fatigue. Among them, the Random Forest model showed superior accuracy and identified greater disability in performing activities of daily living (as indicated by higher HAQ scores), older age, and longer disease duration as key predictors of fatigue in patients with GPA. To further improve prediction accuracy, additional observations and the incorporation of other relevant clinical and demographic variables may be necessary. Furthermore, patients with GPA who experienced fatigue exhibited increased glucocorticoid use and elevated acute-phase reactants, despite comparable disease activity. This dissociation between inflammatory markers and disease activity suggests that fatigue may involve mechanisms beyond overt systemic inflammation, highlighting the need for further investigation. Machine learning appears to be a promising tool for identifying fatigue in clinical settings, particularly in regions with limited access to specialized care.

## Data Availability

The datasets used and/or analyzed during the current study are available from the corresponding author on reasonable request.

## Abbreviations

ACR: American College of Rheumatology
ANCA: Anti-neutrophil cytoplasmic antibodies
AUC: Area under the curve
BMI: Body mass index
BVAS: Birmingham Vasculitis Activity Score
CRP: C-reactive protein
DT: Decision tree
ESR: Erythrocyte sedimentation rate
EULAR: European League Against Rheumatism
GPA: Granulomatosis with polyangiitis
GPA-F: Granulomatosis with polyangiitis with fatigue
GPA-NF: Granulomatosis with polyangiitis without fatigue
HAQ: Health Assessment Questionnaire
IPAC-SF: International Physical Activity Questionnaire - Short Form
LR: Binary logistic regression
MFIS: The Modified Fatigue Impact Scale
ML: Machine learning
ReBEC: Brazilian Clinical Trials Registry
RF: Random forest
UTN: Universal Trial Number
XB: Xtreme Gradient Boosting
